# 

**Published:** 1906-06-09

**Authors:** 


CONTENTS.

The British Medical Association and the General Medical Council - 171

Patent Medicines - 172

Annotations - 173

Medical Opinion and Movement - 174

Hospital Clinics—

The Present Position of Radiation in Treatment - 175

Purulent Pleurisy - 177

Practical Notes on Remedies - 179

Practical Notes on Diagnosis and Treatment - 180

The Book World of Medicine and Science - 181

New Appliances and Things Medical - 181

Medical Appointments and Vacancies - 181

Hospital Administration—

Current Hospital Topics - 182

University College Hospital - 183

Structural and Practical Departments - 183

Additions to the Victoria Infirmary, Glasgow - 184

Hospital Meetings - 185

NURSING SECTION.

Notes on News from the Nursing World—
Death of a Crimean Nurse—Nursing on Steamships—Premiums at Worcester Infirmary—Nurses and Nursing Homes—Difficulties at Plymouth Infirmary—The Nurse in the Almshouse—Quarantine for Puerperal Fever—Working men and District Nursing Associations—Providing for a Temporary Emergency—A New Asylum Matron—Nurses' Missionary League—South Australian Branch of the British Nurses' Association—An Entertainment to Chronic Patients—St. Pancras South Infirmary—Hackney Poor-law Infirmary—The Report of a Medical Inspector - 141-144
The Nursing Outlook - 145
Abdominal Surgery - 146
The Nurse's Clinic - 147
Incidents in a Nurse's Life - 148
Women and Thrift - 149
The Royal National Pension Fund for Nurses - 150
Asylum Workers and Long Hours - 151
The Royal British Nurses' Association - 151
The Middlesex Hospital Nursing and Domestic Reorganisation - 152
Appointments - 152
A Book and Its Story - 15
Notes and Queries - 154
For Reading to the Sick - 154

